# Atypical natural killer T-cell receptor recognition of CD1d–lipid antigens

**DOI:** 10.1038/ncomms10570

**Published:** 2016-02-15

**Authors:** Jérôme Le Nours, T. Praveena, Daniel G. Pellicci, Nicholas A. Gherardin, Fiona J. Ross, Ricky T. Lim, Gurdyal S. Besra, Santosh Keshipeddy, Stewart K. Richardson, Amy R. Howell, Stephanie Gras, Dale I. Godfrey, Jamie Rossjohn, Adam P. Uldrich

**Affiliations:** 1 Infection and Immunity Program & Department of Biochemistry and Molecular Biology, Biomedicine Discovery Institute, Monash University, Clayton, Victoria 3800, Australia; 2 Australian Research Council Centre of Excellence for Advanced Molecular Imaging, Monash University, Clayton, Victoria 3800, Australia; 3 Department of Microbiology and Immunology, Peter Doherty Institute for Infection and Immunity, University of Melbourne, Melbourne, Victoria 3000, Australia; 4 Australian Research Council Centre of Excellence for Advanced Molecular Imaging, University of Melbourne, Melbourne, Victoria 3000, Australia; 5 Cancer Immunology Research Program, Research Division, Peter MacCallum Cancer Centre, East Melbourne, Victoria 3002, Australia; 6 School of Biosciences, University of Birmingham, Edgbaston, Birmingham B15 2TT, UK; 7 Department of Chemistry, University of Connecticut, Storrs, Connecticut 06269-3060, USA; 8 Institute of Infection and Immunity, Cardiff University, School of Medicine, Heath Park, Cardiff CF14 4XN, UK

## Abstract

Crucial to Natural Killer T (NKT) cell function is the interaction between their T-cell receptor (TCR) and CD1d-antigen complex. However, the diversity of the NKT cell repertoire and the ensuing interactions with CD1d-antigen remain unclear. We describe an atypical population of CD1d–α-galactosylceramide (α-GalCer)-reactive human NKT cells that differ markedly from the prototypical TRAV10-TRAJ18-TRBV25-1^+^ type I NKT cell repertoire. These cells express a range of TCR α- and β-chains that show differential recognition of glycolipid antigens. Two atypical NKT TCRs (TRAV21-TRAJ8-TRBV7–8 and TRAV12-3-TRAJ27-TRBV6-5) bind orthogonally over the A′-pocket of CD1d, adopting distinct docking modes that contrast with the docking mode of all type I NKT TCR-CD1d-antigen complexes. Moreover, the interactions with α-GalCer differ between the type I and these atypical NKT TCRs. Accordingly, diverse NKT TCR repertoire usage manifests in varied docking strategies and specificities towards CD1d–α-GalCer and related antigens, thus providing far greater scope for diverse glycolipid antigen recognition.

## Abstract (web summary)

The invariant αβTCR of type I NKT cells recognizes a lipid α-GalCer presented by CD1d. Here the authors describe atypical α-GalCer-reactive NKT cells with diverse TCRs, which bind to CD1d-α-GalCer in a manner distinct from type I NKT cells, thus unveiling greater diversity in lipid antigen recognition.

## Graphical abstract

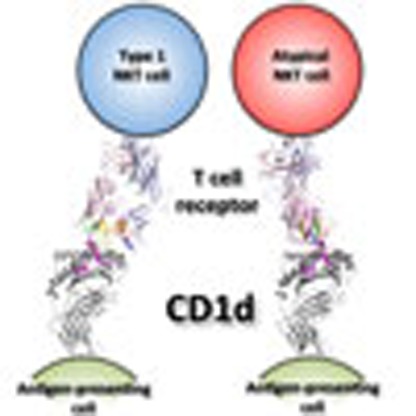

αβ T cells can be activated by peptides, metabolites and lipids when bound to their requisite antigen (Ag)-presenting molecules^1^,^2^,^3^. The CD1 family of MHC-class I-like molecules present an array of endogenous and foreign lipids Ags that are recognized by specialized T-cell populations^4^,^5^. For example, NKT cells are activated by lipid-based Ags presented by CD1d^6^. Based on ligand specificity and αβ TCR composition, NKT cells are broadly sub-divided into two populations, type I and II. α-Galactosylceramide (α-GalCer) is the prototypical Ag for type I NKT cells, which express an invariant TCR α-chain (TRAV10^+^TRAJ18^+^ (Vα24-Jα18) in humans and the orthologous TRAV11^+^TRAJ18^+^ (Vα14-Jα18) in mice)^6^,^7^,^8^. While type II NKT cells display a diverse TCR repertoire, and while their Ag-specificity remains unclear, they are nevertheless characterized as being non-reactive towards α-GalCer^6^,^9^. The apparent functional divergence between type I and type II NKT cells arises, in part, from the interaction between the NKT TCR and CD1d–Ag^8^.

Despite the prototypical type I NKT TCR gene usage, variations within the CD1d–α-GalCer reactive repertoire exist that subsequently impact on ligand specificity and functional outcome. For example, while human type I NKT cells typically use TRBV25-1 (Vβ11)-encoded TCR β-chains, mouse type I NKT cells can utilize TRBV13 (Vβ8), TRBV29 (Vβ7) and TRBV1 (Vβ2) TCR β-chains, with the variations in the TRBV repertoire impacting on the range of ligands a given NKT TCR can interact with^10^,^11^,^12^,^13^,^14^,^15^,^16^. Similarly, both mouse and human NKT cells can utilize alternative TCR α-chains that retains α-GalCer reactivity^17^,^18^,^19^,^20^,^21^. For example, TRAV10^−^TRAJ18^+^TRBV25-1^+^ NKT cells comprise up to 15% of human CD1d–α-GalCer reactive NKT cells^17^. Despite their comparable reactivity to α-GalCer and their identical TRAJ18 usage, these cells appear to exhibit a lower affinity towards α-glucosylceramide (α-GlcCer) compared with the TRAV10^+^TRAJ18^+^ type I NKT cells^17^. In contrast, mouse TRAV13-3^+^TRAJ50^+^TRBV13^+^ (Vα10^+^Jα50^+^Vβ8^+^) CD1d–α-GalCer reactive NKT cells exhibited a greater reactivity towards α-GlcCer in comparison to α-GalCer, and they were selectively reactive to a mycobacterial Ag α-glucuronosyldiacylglycerol^19^. Moreover, a population of α-GalCer-reactive TRDV1^+^ (Vδ1^+^) γδ T cells was identified recently, and these cells also exhibited a distinct lipid–Ag-binding profile, thereby highlighting the breadth of TCR usage that engenders CD1d–α-GalCer recognition^22^. Therefore, if we are to fully understand the significance and therapeutic potential of CD1d–lipid Ag recognition in the immune system, it is vital to understand how variations within the NKT TCR repertoire impacts on CD1d–Ag recognition.

The crystal structures of a large panel of human and mouse type I NKT TCRs have been determined in complex with CD1d presenting a broad repertoire of chemically distinct lipids including synthetic ligands, self- and microbial ligands^10^,^11^,^12^,^15^,^19^,^20^,^23^,^24^,^25^,^26^,^27^,^28^,^29^,^30^,^31^,^32^,^33^,^34^. Universally, despite the NKT cell repertoire and antigenic variations, the resultant type I NKT αβTCR–CD1d–Ag complexes exhibit a highly conserved docking strategy. Namely, the type I NKT TCR docks, in a parallel manner, over the F′-pocket of CD1d^8^. Here the semi-invariant type I NKT TCR α-chain dominated the interaction, binding to CD1d and Ag, whereas the TCR β-chain ligated only to CD1d. Nevertheless, within this consensus footprint, altered contributions from the complementarity determining regions (CDRs) of the NKT TCR led to differing patterns of CD1d–Ag reactivity. For example, the CDR3β loop modulated the extent of CD1d autoreactivity and, hence, the functional response to lipid Ags, despite not contacting the Ag directly^25^,^35^. Moreover, the heightened reactivity of the TRAV13-3^+^TRAJ50^+^ NKT cells towards α-GlcCer was attributable to favourable interactions of the α-GlcCer moiety with the TCR α-chain^19^. While some type II NKT TCRs can dock differently on CD1d, these do not react with α-GalCer and utilize entirely different TCR V genes^36^,^37^. Thus, the question of whether CD1d–α-GalCer-reactive NKT αβTCRs can adopt alternative binding modes that may provide greater diversity in Ag recognition remains to be determined, and this represents an important issue in understanding the scope of lipid Ag recognition by NKT cells.

Here we describe a diverse population of CD1d–α-GalCer reactive cells that we termed ‘atypical NKT cells' because they lack the invariant TRAV10^+^TRAJ18^+^ α-chain and the TRBV25-1 β-chain that are inherent to type I NKT cells. These atypical NKT cells exhibited differing specificities towards lipid Ags compared with that of type I NKT TCRs. Importantly, these atypical NKT cells could respond to glycolipid Ag presented by CD1d with diverse cytokine production, similar to type I NKT cells. Crystal structures of two of these atypical NKT TCRs in complex with CD1d–α-GalCer showed that, in contrast to type I NKT cell TCRs that dock over the F′-pocket of CD1d–α-GalCer, these atypical TCRs docked orthogonally over the A′-pocket of CD1d–α-GalCer. Furthermore, the interactions with the lipid Ag were completely distinct from those observed with type I NKT TCRs engaging α-GalCer-CD1d complexes. Thus, variations in the CD1d–α-GalCer-reactive NKT TCR repertoire can manifest in alternative docking strategies on CD1d and diverse reactivity towards CD1d-restricted lipids.

## Results

### A diverse human type I NKT cell repertoire

**Figure 1:**
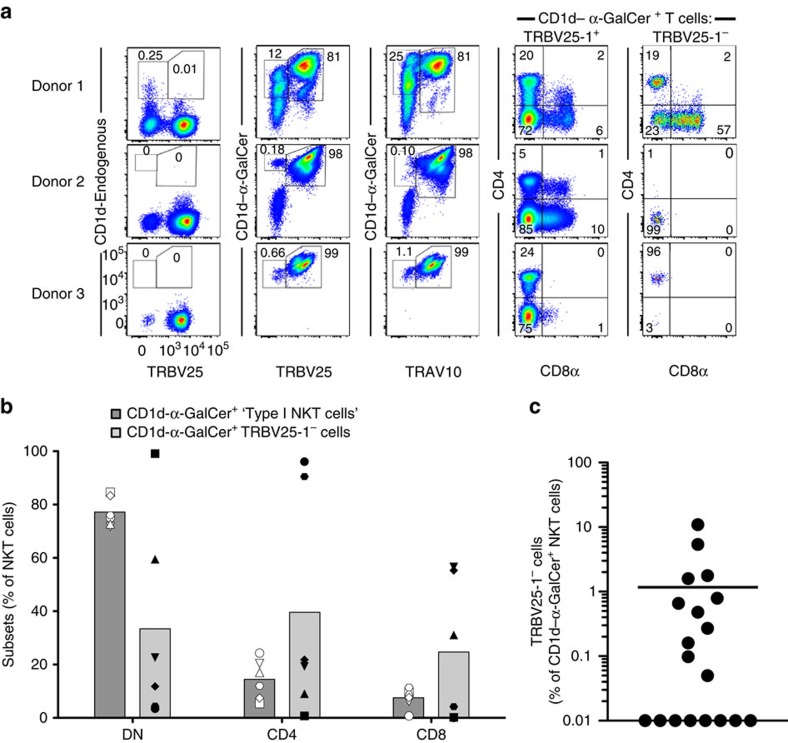
Identification of CD1d–α-GalCer reactive atypical NKT cells. (**a**) Flow cytometry of CD1d–α-GalCer reactive cells enriched and expanded from PBMCs from three healthy human donors. TRDV1^−^ γδTCR^−^ cells were analysed for the expression of TRBV25-1 versus CD1d-endogenous tetramer or CD1d–α-GalCer tetramer (left-hand density plots). TRDV1^−^ γδTCR^−^ CD1d–α-GalCer tetramer^+^ cells were analysed for the expression of TRAV10 (middle density plots). CD1d–α-GalCer tetramer^+^ TRBV25-1^+^ type I cells and CD1d–α-GalCer tetramer^+^ TRBV25-1^−^ cells were analysed for the expression of CD4 and CD8α (right-side density plots). (**b**) The mean percentage of double negative (DN), CD4^+^ and CD8^+^ cells among CD1d–α-GalCer tetramer^+^ TRBV25^+^ Type I cells (dark grey) and CD1d–α-GalCer tetramer^+^ TRBV25^−^ cells (light grey). Each symbol represents cells from a different donor (*n*=6). (**c**) The mean percentage of CD1d–α-GalCer tetramer^+^ TRBV25^−^ cells of total CD1d–α-GalCer reactive NKT cells, from 19 individual donors. Donors that showed no clear population of atypical NKT cells were given an arbitrary value of 0.01%.

A defining characteristic of type I NKT cells is their reactivity towards the prototypical type I NKT cell Ag, α-GalCer, presented by CD1d^6^. The human type I NKT cell repertoire is comprised of the invariant TRAV10^+^TRAJ18^+^TRBV25-1^+^ NKT cells. Given that a range of TCR β-chains can support CD1d–α-GalCer recognition in mice^8^, we were interested in exploring whether a similar population of TRBV25-1^−^ NKT cells existed in humans. To establish this, we isolated and expanded CD1d–α-GalCer reactive NKT cells from healthy human blood donors and performed analytical flow cytometry to identify non-canonical NKT cell TCR subsets, by staining with antibodies specific for TRAV10 and TRBV25-1, along with γδTCR and TRDV1 to exclude CD1d–α-GalCer-reactive γδ T-cells from the analysis^22^. Using this approach, we detected a clear population of TRBV25-1^−^ NKT cells, that, in most donors, did not react with ‘endogenous' CD1d tetramers, thus implying these cells recognized α-GalCer presented by CD1d (Fig. 1a). Consistent with earlier studies^17^,^38^, a population of TRAV10^−^ NKT cells was also detected, although interestingly, the proportion of the TRBV25-1^−^ and the TRAV10^−^ populations within each sample did not always coincide, suggesting that these two subsets were at least partially mutually exclusive. Co-staining with CD4 and CD8α co-receptors revealed a variable pattern of expression on the CD1d–α-GalCer-restricted TRBV25-1^−^ cells compared with type I NKT cells (Fig. 1a,b). A more extensive phenotypic analysis of four donors with a detectable population of these cells indicated that while both TRBV25-1^+^ (type I) and TRBV25-1^−^ CD1d–α-GalCer-reactive cells expressed NKG2D (three out of four donors each), there was very little or no expression of a panel of killer inhibitory receptors (including CD158A/B/F/G/H) or CD56 (Supplementary Fig. 1). Furthermore, CD161 was clearly expressed on the TRBV25-1^−^ cells in two out of four donors, versus three out of four donors for type I NKT cells. Thus, while there appears to be significant heterogeneity in the phenotypic profiles of TRBV25-1^−^ cells between donors, at least in some cases they resemble type I NKT cells. Analysis of additional donors confirmed that the γδTCR^−^ TRBV25-1^−^ subset was clearly detectable in 11/19 individuals, where they ranged from 0 to 10% of all CD1d–α-GalCer reactive (type I) NKT cells (Fig. 1c). Given that type I NKT cells generally represent ∼0.01–0.1% of peripheral blood lymphocytes, this indicates that these cells are normally quite rare, and in most cases, we could only readily detect them after *in vitro* enrichment/expansion of CD1d–α-GalCer-reactive cells.

To determine the paired TCR α and β chain usage of these cells, we performed single-cell TCR sequencing and compared gene usage with TRAV10^+^TRBV25-1^+^ type I NKT cells sorted from the same donors. The sequencing results confirmed that there appeared to be two distinct subsets of non-canonical CD1d–α-GalCer-reactive T cells based on TCR gene usage. One of these exhibited a TRAV10^−^TRBV25-1^+^ phenotype, and was only identified within the TRAV10^−^ population, whereas the second was TRAV10^−^TRBV25-1^−^, and was present within both the TRAV10^−^ and TRBV25-1^−^ populations (Table 1). Interestingly, these data also revealed a close association between TRAJ18 and TRBV25-1 gene usage. For example, eight out of eight unique TRBV25-1^+^ TCR sequences that lacked the TRAV10 TCR α-chain still expressed TRAJ18. In contrast, only one out of fourteen unique TCR sequences that were TRBV25-1^−^ utilized TRAJ18 (*P*<0.0001; Fisher's exact test). Instead, this TRBV25-1^−^ population displayed a broad spectrum of TRAV and TRAJ gene usage (Table 1). Thus, from TRBV25-1^−^ cells, 13 different TCR α-chains utilized TRAV genes other than TRAV10 and these paired with a range of TRAJ genes, including TRAJ8, 24, 27, 30, 44, 48 and 52. These TRAJ segments displayed very limited sequence identity with the TRAJ18 gene segment, and minimal inter-sequence or inter-donor similarity (Table 1). The CDR1α and CDR2α loops displayed notable sequence variability, and moreover, on account of variable N-region additions and deletions, the length of the CDR3α loop varied from 10 to 14 amino acids among the TRBV25-1^−^ subset, compared with the highly restricted CDR3α of both the TRAV10^−^ TRBV25-1^+^ and TRAV10^+^TRBV25-1^+^ subsets, where 8/8 and 11/11 clones, respectively, had an invariant CDR3α length of 13 residues (Table 1). In addition, the TCR β-chain gene usage was highly diverse, and included TRBV2, 4-1, 6-5, 7–8, 11-2, 12-5, 20-1 and 28, along with diverse CDR3β sequence and length (Table 1), and no identical clones were identified between separate donors. Accordingly, the CD1d–α-GalCer-reactive T-cell compartment is not only comprised of dominant TRAV10^+^TRAJ18^+^TRBV25-1^+^ and sub-dominant TRAV10^−^TRAJ18^+^ TRBV25-1^+^ ‘public' repertoires, but in many cases, it also includes diverse TRAV10^−^TRAJ18^−^TRBV25-1^−^ ‘private' TCR repertoires. We refer to these latter cells as ‘atypical' NKT cells.

### Differing patterns of Ag reactivity

**Figure 2:**
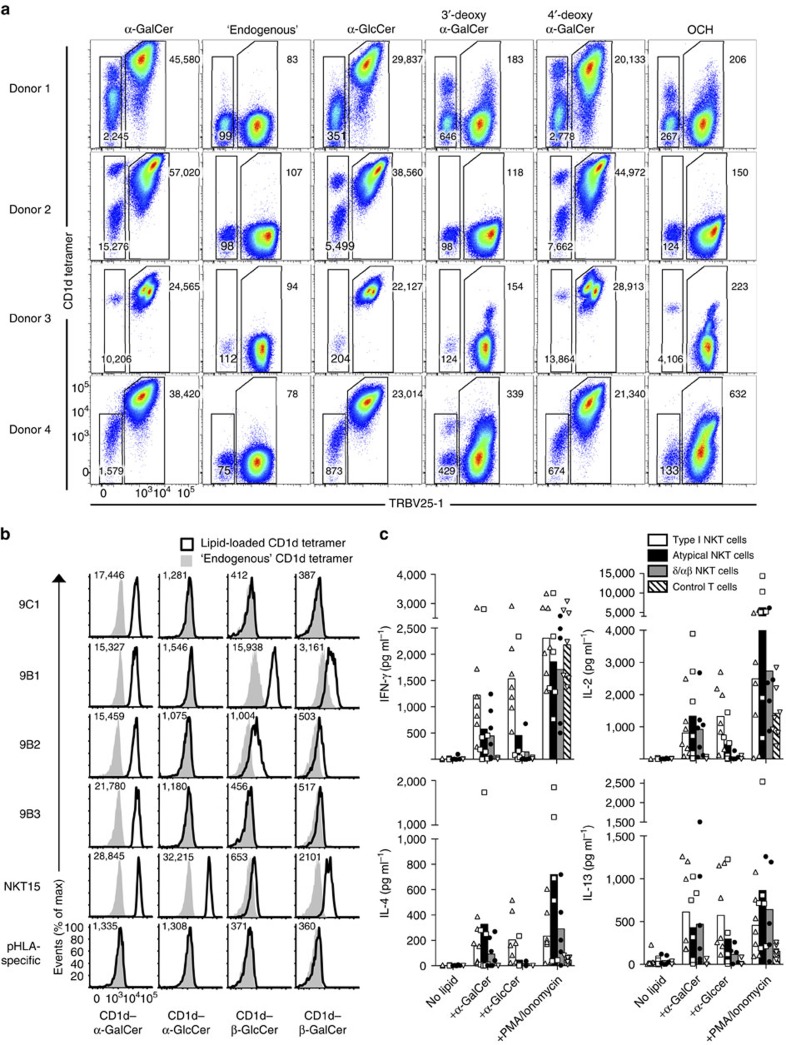
Lipid reactivity of atypical TRBV25-1^−^ NKT cell lines. (**a**) CD1d tetramer staining of CD1d–α-GalCer-reactive cells enriched and expanded from PBMCs from four healthy human donors. Plots show TRBV25-1 versus CD1d tetramers loaded with α-GalCer (C24:1), ‘endogenous' antigen, α-GlcCer, 3′-deoxy-α-GalCer, 4′-deoxy-α-GalCer or OCH. Data show one of two representative experiments. (**b**) Histograms depicting human CD1d–lipid antigen tetramer staining (white histograms) of CD3^+^ Jurkat T-cell lines transduced with the 9C1, 9B1, 9B2, 9B3 atypical NKT cell TCRs or with the NKT15 type I NKT cell TCR or an irrelevant pHLA-specific TCR control, overlaid with ‘endogenous' tetramers (grey histograms). Numbers in each histogram represent CD1d–lipid tetramer mean fluorescence intensity. Data are representative of two separate experiments. (**c**) Graphs depict the mean IFN-γ, IL-2, IL-4 and IL-13 concentrations in culture supernatants of 4–5 × 10^3^ *in vitro*-expanded/purified CD1d–α-GalCer tetramer^+^ TRBV25-1^+^ (type I NKT, white bars), CD1d–α-GalCer tetramer^+^ TRBV25-1^−^ (atypical NKT, black bars), CD1d–α-GalCer tetramer^+^ TRDV1^+^ γδTCR^−^ (δ/αβ NKT, grey bars), and CD1d–α-GalCer tetramer^−^ (control T cells, hashed bars), with different lipid Ag (0.5 μg ml^−1^) in the presence of K562.CD1d APCs or PMA/ionomycin for 24 h. Data are representative of *n*=5–7 donors, with each symbol depicting a separate donor (each symbol derived from *n*=1–2 technical replicates). Data are pooled from two independent experiments.

A feature of the type I NKT TCR is that it not only reacts with α-GalCer, but also imbues reactivity to a range of other self and foreign ligands. To establish the Ag-reactivity profile of atypical NKT cells, we stained CD1d–α-GalCer tetramer-enriched and expanded PBMC samples from healthy donors with a panel of CD1d–Ag tetramers, and compared the TRBV25-1^+^ type I and TRBV25-1^−^ atypical NKT cells within each donor. While all type I NKT cells bound to α-GalCer and α-GlcCer-loaded CD1d, many of the atypical NKT cells failed to stain with α-GlcCer-loaded CD1d tetramers (Fig. 2a). As we have previously published^12^, human type I NKT cells exhibited a strong dependence on the 3′-OH moiety of α-GalCer as evidenced by their lack of reactivity to 3′-deoxy α-GalCer. However, subsets of atypical NKT cells in donors 1 and 4 clearly tolerated this substitution (Fig. 2a). Differences between the Ag-reactivity profile of type I and atypical NKT cells were also evidenced using the OCH analogue of α-GalCer, which has a truncated sphingosine chain. While this analogue is only poorly recognized by type I NKT cells, some subsets of atypical NKT cells, such as those in donor 1 and donor 3, still recognized this Ag (Fig. 2a). Thus, the diverse TCR expression by atypical NKT cells facilitates an altered and mixed pattern of CD1d–Ag reactivity compared with type I NKT cells.

To confirm the non-canonical TRBV25-1^−^ atypical NKT cell TCRs (Table 1) were indeed CD1d-restricted, we generated a panel of Jurkat T-cell lines transduced with TRBV25-1^−^ TCRs and examined their ability to bind CD1d tetramers loaded with a range of lipid Ags. We selected four TRBV25-1^−^ TCRs that represented a cross-section of the TCRα and TCRβ chain usage: clones 9C1 (TRAV21^+^TRAJ8^+^TRBV7–8^+^; Table 1, sequence #13); 9B1 (TRAV38-1^+^TRAJ48^+^TRBV9; Table 1, sequence #1); 9B2 (TRAV12-3^+^TRAJ27^+^TRBV6-5^+^; Table 1, sequence #2); 9B3 (TRAV13-2^+^TRAJ24^+^TRBV20-1^+^; Table 1, sequence #3) and two controls, namely a Jurkat pHLA-specific irrelevant TCR (TRAV17^+^TRBV16^+^) and an SKW3 TRAV10^+^TRAJ18^+^TRBV25-1^+^ type I NKT TCR^+^ cell line (SKW3.NKT15) (Fig. 2b). As expected, the pHLA-specific TCR did not bind to CD1d–Ag, while the SKW3.NKT15 cell line bound to CD1d–α-GalCer, but not CD1d tetramer loaded with endogenous Ags (Fig. 2b). The 9C1, 9B1, 9B2 and 9B3 Jurkat cell lines all bound to the CD1d–α-GalCer tetramer, but not to CD1d-endo, thereby confirming the CD1d-restriction and α-GalCer reactivity of these TCRs isolated from human PBMCs (Fig. 2b). While the SKW3.NKT15 cell line could readily bind CD1d–α-GlcCer, the atypical NKT TCRs did not tolerate this substitution, suggesting clear differences in how the atypical NKT TCRs interacted with the glycosyl headgroup compared with type I NKT TCRs (Fig. 2b). Similar to the trends in Fig. 2a, these cell lines exhibited a differential pattern of reactivity to the α-GalCer analogues 3′-deoxy-α-GalCer, 4′-deoxy-α-GalCer and OCH (Supplementary Fig. 2).

Type I NKT cells can recognize β-linked self-ligands by moulding these ligands into a structural conformation resembling their α-linked counterparts, albeit with reduced affinity^25^. Notably, in contrast to the type I SKW3.NKT15 cell line, we detected clear reactivity of Jurkat.9B1 and Jurkat.9B2 cell lines to β-GlcCer (Fig. 2b). This β-GlcCer reactivity, in the absence of α-GlcCer reactivity, is an indication that these atypical NKT TCRs were not reacting with any potential α-GlcCer contamination within the β-GlcCer preparation^39^. These 9B1 and 9B2 TCRs also reacted to a lesser extent with β-GalCer (Fig. 2b), but none reacted with the type II NKT cell ligand sulfatide or the ganglioside GD3 (Supplementary Fig. 2). In addition, and in contrast to human type I NKT cells, there was no cross-species reactivity of any of these atypical NKT TCRs TCRs towards mouse CD1d–α-GalCer (Supplementary Fig. 2). Therefore, non-canonical TRBV25-1^−^ NKT cell TCRs are capable of recognizing a diverse array of both α- and β-linked lipid Ags, with a spectrum and hierarchy of reactivity that is distinct from typical type I NKT cells.

To test the Ag responsiveness of atypical NKT cells, we isolated CD1d–α-GalCer tetramer^+^ TRBV25-1^−^ cells from PBMC by flow cytometric sorting, and then *in vitro*-expanded these cells with anti-CD3/CD28 in the presence of irradiated allogeneic PBMC for 2 weeks. Using this approach, seven out of eight donors had a clear population of atypical NKT cells after expansion, as well as the recently described CD1d–α-GalCer tetramer^+^ TRDV1^+^ δ/αβ NKT cells^40^ (Supplementary Fig. 3), with typical yields of ∼10^4^–10^6^ cells for each subset per donor, after expansion. We re-sorted these populations, along with type I NKT cells (CD1d–α-GalCer tetramer^+^ TRBV25-1^+^) and control (CD1d–α-GalCer tetramer^−^) T cells derived from the same cultures, and measured cytokine production after a 24 h challenge with different lipid Ag in the presence of CD1d-expressing APCs. Consistent with their tetramer reactivity, atypical NKT cells from five out of seven donors elicited a clear response following challenge with α-GalCer, producing both Th1- (IFN-γ, IL-2) and Th2- (IL-4, IL-13) type cytokines, compared with control cultures containing APCs alone (Fig. 2c). Both type I (seven out of seven donors) and δ/αβ (six out of seven donors) NKT cells responded in a similar fashion, however as expected, the control T cells did not respond to any lipid Ag (none of the seven donors) despite responding to PMA/ionomycin. Consistent with the tetramer-staining patterns, most atypical NKT cells exhibited reduced reactivity to α-GlcCer compared with type I NKT cells. Thus, these data confirm that atypical NKT cells can respond to glycolipid Ag presented by CD1d with diverse cytokine production, similar to type I NKT cells.

**Figure 3:**
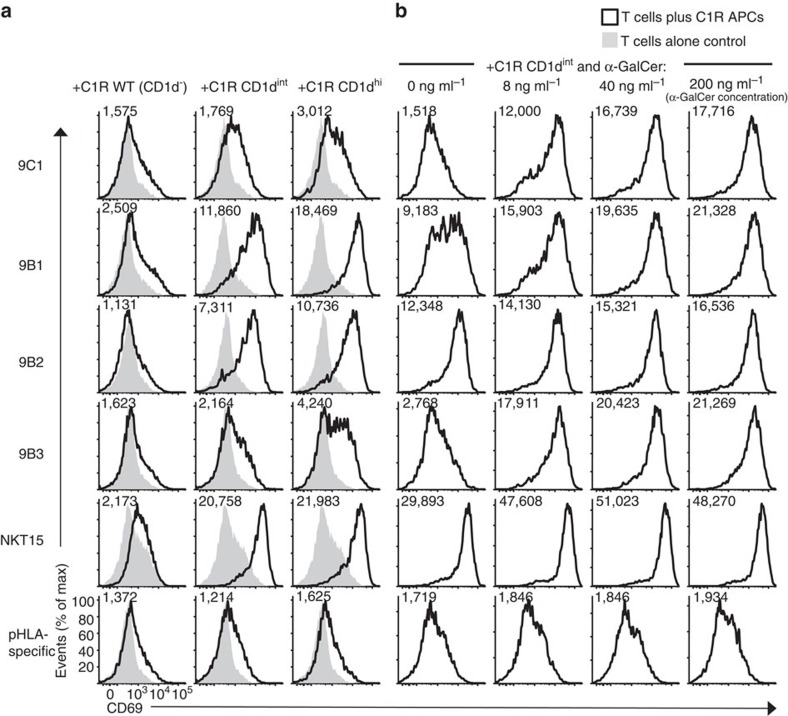
Functional reactivity of atypical TRBV25-1^−^ NKT cell lines to CD1d–lipid Ag. (**a**) Histograms depicting CD69 expression on gated Jurkat T-cells lines transduced with the 9C1, 9B1, 9B2, 9B3, NKT15 or control pHLA-specific TCRs, after overnight *in vitro* co-culture with either WT (CD1d^−^), CD1d-intermediate (CD1d^int^) or CD1d-high (CD1d^hi^)-expressing C1R APCs (left-hand columns), or (**b**) with CD1d^int^ C1R APCs plus graded concentrations of α-GalCer (C_26:0_) (right-hand columns). Numbers in each histogram represent CD69 mean fluorescence intensity (MFI). Data in **a** (three left-hand columns) are representative of two separate experiments; data in **b** (right-hand columns) are each representative of one experiment.

Next, using CD69 upregulation as a marker of functional activation, we examined the ability of the transduced Jurkat.NKT cell lines to be activated in the presence of C1R cells expressing CD1d plus defined Ag. Following overnight co-culture with C1R cells expressing intermediate or high levels of CD1d, but not CD1d^−^ C1R WT cells, all the atypical Jurkat.NKT cell lines showed clear signs of activation (Fig. 3a). This was despite no obvious binding to the CD1d-endogenous tetramers (Fig. 2b), suggesting the activation assays were more sensitive than the tetramer-based assays. This also confirmed that these atypical NKT TCRs are capable of initiating cellular activation following TCR ligation by CD1d–Ag. Addition of graded concentrations of α-GalCer to these co-cultures resulted in greater activation of the Jurkat.9C1, 9B1 and 9B3 cell lines, yet only appeared to marginally enhance activation of the Jurkat.9B2 cell line (Fig. 3b). Thus, these data show that non-canonical TRBV25-1^−^ atypical NKT cell TCRs confer functional reactivity to CD1d, but they also demonstrate diverse and distinct patterns of Ag reactivity compared with TRAV10^+^TRAJ18^+^ type I NKT cells.

### Affinity towards CD1d–Ag

**Figure 4:**
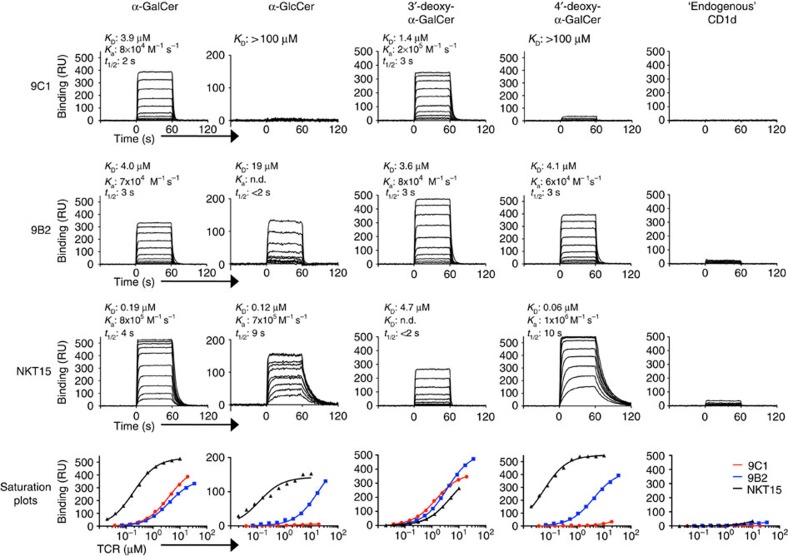
Affinity of non-canonical TRBV25-1^−^ NKT cell TCRs to CD1d–Ag. The affinity of TCR-CD1d–Ag interactions were determined by surface plasmon resonance, by measuring the binding of graded concentrations of soluble 9C1 (19–0.038 μM), 9B2 (34–0.067 μM), and a type I NKT cell control (NKT15, 10–0.02 μM), to human CD1d loaded with α-GalCer, α-GlcCer, 3′-deoxy α-GalCer, 4′-deoxy α-GalCer or CD1d-endogenous. Saturation plots for 9C1 (red), 9B2 (blue) and NKT15 (black) versus each respective ligand are shown in the lower panels. *K*_D_, dissociation constant; *K*_a_, association rate; *t*_1/2_, half-life. Results are representative of two similar experiments.

Using surface plasmon resonance (SPR), we next determined the affinity of two atypical NKT TCRs from clones 9C1 and 9B2, towards CD1d bound to α-GalCer and variants thereof. These two TCRs were selected based on their contrasting Ag reactivity profiles, whereby the 9C1 TCR demonstrated a strong dependence on α-GalCer for activation, whereas the 9B2 TCR, whilst still reactive to CD1d–α-GalCer tetramers, demonstrated an auto-reactive profile that was associated with less Ag-specific activation. We expressed, refolded and purified the soluble ectodomains of both TCRs to high yields, and passed them over CD1d–Ag coupled to a sensor chip. The 9C1 and 9B2 TCRs did not bind, or bound very poorly, to CD1d-endogenous tetramers respectively, consistent with the tetramer-binding data (Fig. 4). The 9C1 TCR and 9B2 TCR both bound to CD1d–α-GalCer with an affinity (*K*_D_) of 3.9 μM and 4.0 μM respectively, values that, while comparable to many TCR–pMHC interactions, were weaker than the affinity of the canonical type I NKT TCR (NKT15) towards CD1d–α-GalCer (*K*_D_=0.19 μM) (Fig. 4). The affinity of the 9C1 and 9B2 TCRs towards CD1d-3′-deoxy-α-GalCer (*K*_D_=1.4 μM and 3.6 μM, respectively) was comparable or moderately higher than that of CD1d–α-GalCer (Fig. 4). This is in stark contrast to the NKT15 TCR, which bound with much lower affinity to CD1d-3′-deoxy-α-GalCer (*K*_D_=4.7 μM, ∼20-fold reduction) (Fig. 4)^12^,^26^. Conversely, the 9C1 TCR exhibited a markedly reduced affinity (*K*_D_>100 μM) towards the 4′-deoxy-α-GalCer analogue, while there was no negative impact of this analogue on NKT15 or 9B2 TCR binding (Fig. 4). Consistent with tetramer staining and functional studies, α-GlcCer was bound with much lower affinity by 9C1 TCR and 9B2 TCR (*K*_D_>100 μM and 19 μM, respectively), yet was well-tolerated by NKT15 TCR (*K*_D_=0.12 μM) (Fig. 4). The recognition of 4′-deoxy-α-GalCer but not α-GlcCer by 9B2 TCR implies that the equatorial 4′-OH group of α-GlcCer may cause a conformational change in CD1d and/or the lipid headgroup, which is not tolerated by 9B2. Thus, while the atypical NKT TCRs and type I NKT TCRs are reactive towards α-GalCer, they clearly differ in their fine specificity towards CD1d-restricted Ags.

### Overview of atypical NKT TCR ternary complexes

**Figure 5:**
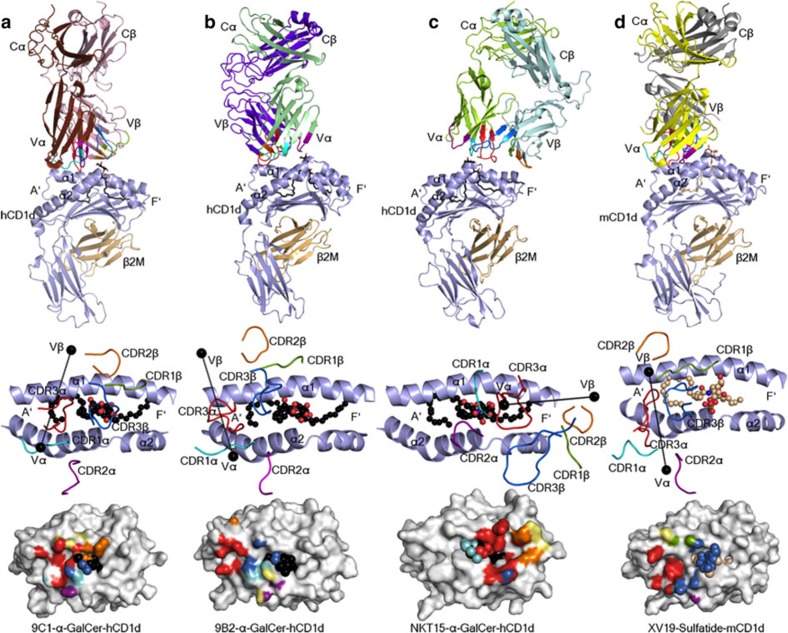
Overview of the docking of atypical NKT TCR ternary complexes. Ternary complexes of (**a**) human 9C1 TCR–CD1d–α-GalCer, (**b**) 9B2 TCR–CD1d–α-GalCer, (**c**) NKT15 TCR–CD1d–α-GalCer (PDB code 2PO6 (ref. 27) and (**d**) mouse XV19 TCR–CD1d–sulfatide (PDB code 4EI5 (ref. 36). Top panels depict an overview of each structure, middle panels illustrate the TCRs docking onto CD1d and lower panels show the TCR footprints on the CD1d–Ag molecular surface. The CD1d and β2-microglobulin molecules are coloured in light blue and light brown, respectively. 9C1 TCRα, brown; 9C1 TCRβ, light pink; 9B2 TCRα, light green; 9B2 TCRβ, purple; NKT15 TCRα, green; NKT15 TCRβ, cyan; XV19 TCRα, yellow; XV19 TCRβ, grey. The CDR loops are coloured as follows: CDR1α, aqua; CDR2α, purple; CDR3α, red; CDR1β, green; CDR2β, orange; CDR3β, blue. The α-GalCer and sulfatide are coloured in black and light brown sticks (top panel), or black and light brown spheres (middle and lower panels), respectively. In the middle panels, the centre of mass of the respective TRAV and TRBV variable domains are shown as black spheres. In the bottom panels, the molecular surface of CD1d is coloured in light grey.

**Figure 6:**
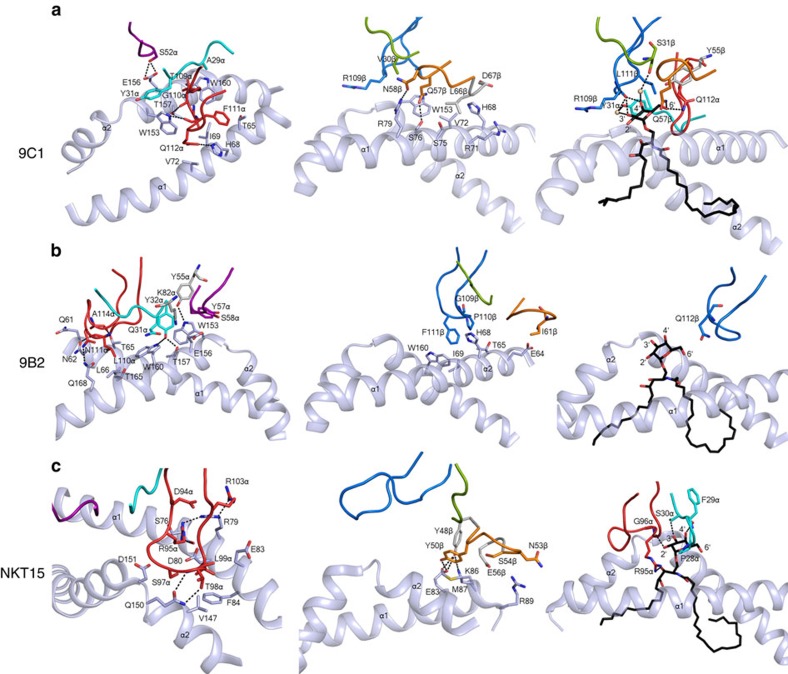
Interactions at the CD1d–Ag–TCR interface. (**a**) Left panel, 9C1 TCR α-chain interactions with CD1d; middle panel, 9C1 TCR β-chain interactions with CD1d; right panel, 9C1 TCR interactions with α-GalCer; (**b**) Left panel, 9B2 TCR α-chain interactions; middle panel, 9B2 TCR β-chain interactions with CD1d; right panel, 9B2 TCR interactions with α-GalCer; (**c**) Left panel, NKT15 TCR α-chain interactions with CD1d; middle panel, NKT15 TCR β-chain interactions with CD1d; right panel, NKT15 TCR interactions with α-GalCer. For clarity, only the hydrogen bonds are shown as black dashed lines and the α1- and α2-helices of CD1d are shown as cartoon representation and coloured in light blue. CDR loops are coloured according to Fig. 5; spheres represent water molecules.

Next, to establish how atypical NKT TCR usage manifested in CD1d–Ag recognition, we determined the crystal structures of the 9C1 TCR-CD1d–α-GalCer and 9B2 TCR-CD1d–α-GalCer ternary complexes to 2.5 and 3.1 Å resolution, respectively (Supplementary Tables 1–3, Supplementary Fig. 4). The 9C1 (Fig. 5a) and 9B2 (Fig. 5b) TCRs adopted two distinct docking modes atop CD1d–α-GalCer, both of which markedly contrasted the salient parallel docking mode over the F′-pocket that is observed for NKT15 TCR-CD1d–α-GalCer (Fig. 5c) and all other type I NKT TCR–CD1d–Ag complexes determined to date^8^. The 9C1 TCR docked orthogonally (75°) across the A′-pocket of CD1d, in which the 9C1 TCR α-chain was located above the CD1d α2-helix, while the TCR β-chain was more centrally positioned over the CD1d α1-helix (Fig. 5a). On ligation, the buried surface area (BSA) of the 9C1 TCR was ∼750 Å^2^, whereupon the TCR α-chain contributed the most (∼460 Å^2^) to the 9C1 TCR-CD1d–α-GalCer interface (Fig. 5a). We also determined the structure of the 9C1 TCR in the non-liganded state (Supplementary Table 1), thereby allowing us to compare the mode, and plasticity of atypical NKT TCR recognition to that of typical type I TCR recognition of CD1d–α-GalCer (Figs 5 and 6). The 9C1 TCR did not undergo a major structural rearrangement on CD1d–α-GalCer engagement, although the CDR3 loops moved to bind the α-GalCer moiety (Supplementary Fig. 4d). The 9B2 TCR also sat over the A′-pocket of CD1d with a docking angle of ∼110° across the Ag-binding cleft (Fig. 5b). Thus, while the overall position of the 9B2 TCR α-chain was similar to the 9C1 TCR α-chain atop CD1d (centre of mass (COM) difference of 0.5 Å) (Supplementary Fig. 4c), the 9B2 TCR β-chain was located more towards the extreme end of the CD1d A′-pocket, with a COM difference of 13 Å and rotational difference of 35° compared with the 9C1 TCR β-chain (Fig. 5b and Supplementary Fig. 4c). In comparison, the BSA at the 9B2 TCR-CD1d–α-GalCer interface was 720 Å^2^ (Fig. 5b). The docking mode of these atypical NKT TCRs were more analogous to the mouse type II NKT cell XV19 TCR–CD1d–sulfatide ternary complex (Fig. 5d), although consistent with the definition of type II TCRs, XV19 fails to interact with CD1d–α-GalCer^36^,^37^. Accordingly, this represents the first description of how variations in NKT TCR usage can manifest in a markedly different binding mode towards CD1d–α-GalCer.

### Atypical NKT TCR interactions with CD1d

In the 9C1 TCR–CD1d–α-GalCer ternary complex, the CDR3α loop (35% BSA) played a principal role in the interactions (Fig. 5a). Notably, the characteristics and conformation of the TRAJ8-encoded CDR3α loop of the 9C1 TCR contrasted that of the polar-rich TRAJ18-encoded CDR3α loop of the NKT15 TCR (Table 1). The CDR1α and CDR2α loops of the 9C1 TCR exclusively contacted the CD1d α2-helix, with Ser52α hydrogen bonding to Glu156, the aliphatic moiety of which contacted Tyr31α (Fig. 6a left panel, Supplementary Table 2). Trp153 of CD1d also packed against Tyr31α, and nestled against the CDR3α loop, forming van der Waals (vdw) contacts with its main chain as well as Gln112α (Fig. 6a, left panel). Here, the CDR3α loop contacted residues from the α2-helix (spanning from Trp153–Trp160), and the α1-helix (spanning Thr65–Val72). As such, the CDR3α was wedged within the Ag-binding cleft, with Thr109α stacking against Trp160 and hydrogen bonding to Thr157 of CD1d, while Gln112α formed vdw contacts with Val72 (Supplementary Table 2) and hydrogen bonded to His68 of CD1d (Fig. 6a, left panel). The interactions between the 9C1 TRBV7–8-encoded TCR β-chain and CD1d were more limited, being largely dominated by the CDR2β (BSA 14%) and neighbouring framework regions ligating to the α1-helix of CD1d. Here Gln57β and Asn58β hydrogen bonded to Ser76 and Arg79 of CD1d, respectively, while Leu66β packed against Val72 (Fig. 6a, middle panel).

**Figure 7:**
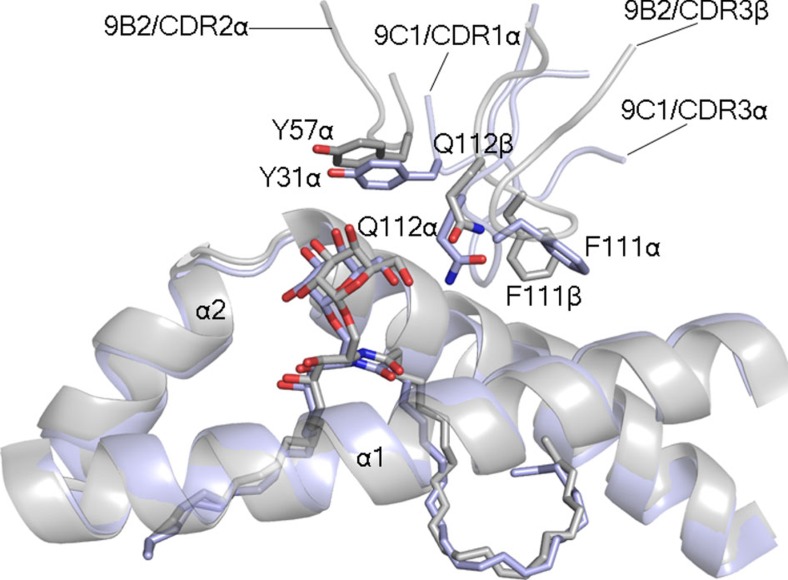
Molecular mimicry between the 9B2 and 9C1 TCR-CD1d–α-GalCer complexes. Superposition of the 9B2 and 9C1 TCR ternary complexes, coloured in grey and light blue, respectively. The superposition is based on the CD1d molecules of each complex. For clarity, only the CDR1α/CDR3β of 9C1 and the CDR2α/CDR3α 9B2 are shown.

In the 9B2 TCR ternary complex, the TCR α-chain chain mediated most of the interactions with CD1d–α-GalCer (BSA 64%), within which the CDR3α loop, the characteristics of which are also distinct from the TRAJ18-encoded CDR3α loop, was the principal contributor to the interface (32% BSA) (Figs 5b, 6b left panel). The CDR1α (BSA 15%) and CDR2α (BSA 12%) made exclusive contacts with the α2-helix of CD1d, whereupon Tyr32α wedged between Trp153 and Trp160 and hydrogen bonded to Thr157 and Trp160; Trp160 also packed against Gln31α (Fig. 6b left panel). Trp153 of CD1d also stacked against Tyr57α, which occupied the same location as Tyr31α from the CDR1α loop of the 9C1 TCR (Fig. 7). The CDR2α loop interactions were enhanced by the neighbouring framework residue, Lys82α, salt-bridging to Glu156 of CD1d (Supplementary Table 3). Central to the CDR3α loop-mediated contacts was Leu110α, which sat within the central axis of the Ag-binding cleft and formed vdw contacts with Asn62, Leu66, Trp160 and Thr165 (Fig. 6b, left panel and Supplementary Table 3). Supplementing these interactions was Ala114α, which was packed against the α1-helix and the main chain carbonyls of Leu110α, and Ala114α forming hydrogen bonds with Asn62 and Thr65 of CD1d, while Asn111α hydrogen bonded to Gln168 (Fig. 6b). Regarding the 9B2 TCR β-chain interactions, the CDR3β loop was the principal contributor to this interface (BSA 20%), as the CDR1β and CDR2β loops played lesser roles (7 and 5% BSA respectively). Here Tyr31β, Val57β and Ile61β aligned to form a focused interaction site spanning residues 64–68 on the α1-helix of CD1d (Supplementary Table 3). The CDR3β loop was positioned between the α1- and α2-helices, where Phe111β plugged a hydrophobic-lined cavity formed by Thr65, His68, Ile69 and Trp160 (Fig. 6b, middle panel). Notably, Phe111β and Gln112β of the 9B2 TCR mirrored the position of Phe111α and Gln112α, respectively, from the 9C1 TCR (Figs 6b and 7). In both 9C1 TCR and 9B2 TCR ternary complexes, the three CDRα loops and the CDR3β are involved in mediating the CD1d interactions. This is in clear contrast to the classical NKT15 type I ternary complex, whereby only the CDR3α contacted the CD1d molecule while the CDR3β was not involved in any interactions with CD1d (Fig. 6c, left and middle panels). Interestingly, while there were notable differences in the sequences of the 9C1 and 9B2 TCRs and respective interatomic TCR-CD1d contacts, there was nevertheless a degree of focused structural mimicry within these atypical type I NKT TCR–CD1d–α-GalCer ternary complexes (Fig. 7). Thus, atypical and type I NKT TCRs engaged CD1d–α-GalCer in a markedly different manner.

### Interactions with α-GalCer

In both the 9C1 and 9B2 TCR ternary complexes, the electron density for α-GalCer was unambiguous (Supplementary Fig. 4a,b). While the positioning of α-GalCer was very similar within the ternary complexes of the atypical NKT TCR and the type I NKT TCR complexes, the ensuing interactions with the lipid Ag were markedly different. In the 9C1 TCR ternary complex, both the α- and β-chains mediated lipid Ag recognition, with direct interactions arising from the CDR3α, CDR2β and CDR3β loops. To enable this, the CDR3β residues (Ser108β, Arg109β, Asp110β and Leu111β) and Gln112α in the CDR3α rearranged to accommodate the Ag (Supplementary Fig. 4d). Here, the main chain carbonyl of Arg109β hydrogen bonded to the 4′-OH of α-GalCer, the latter of which also contacted Ser31β via a water-mediated hydrogen bond (Fig. 6a, right panel). Further, a water-mediated hydrogen bond between the 3′-OH and Tyr31α was observed. The 6′-OH hydrogen bonded to Gln112α and Gln57β (Fig. 6a) and interacted with the framework residue Tyr55β (Supplementary Table 2). While, in the 9B2 TCR ternary complex, the interactions with the α-GalCer moiety were extremely limited, namely, Gln112β solely contacted the 6′-OH of α-GalCer (Fig. 6b, right panel). Both these atypical NKT TCR–α-GalCer contacts contrasted with that of the type I NKT TCR ternary complex. Here interactions with α-GalCer were mediated only via the type I NKT TCR α-chain, where the 2′-OH, 3′-OH and 4′-OH groups are closely sequestered by the CDR1α and CDR3α loops, while the 6′-OH moiety was solvent exposed (Fig. 6c, right panel).

Given the fundamental differences in the contacts with Ag, we probed the importance of the 9C1 and 9B2 TCR residues that contacted the α-GalCer moiety. To establish this, we undertook a mutagenesis/SPR approach on the 9C1 and 9B2 TCRs. For the 9C1 TCR, this included analysing the impact of nine mutants: Tyr31αAla, Tyr31αPhe, Gln112αAla, Ser31βAla, Tyr55βAla, Tyr55βPhe, Gln57βAla, Arg109βAla and Leu111βAla, while for the 9B2 TCR, this involved Gln112βAla mutant only (Table 2). For the 9C1 TCR, while the Ser31βAla mutant had no effect, mutations of the residues contacting the 6′-OH of α-GalCer, namely, Tyr55βAla, Tyr55βPhe, Gln112αAla and Gln57βAla impacted on the binding affinity relative to the wild-type 9C1 TCR. Although Leu111β interacted with the C6 and 4′-OH of the Ag via vdw contacts, the Leu111βAla mutation completely ablated CD1d–α-GalCer recognition. The effect of this mutant may be attributable to the major role Leu111β plays in contacting CD1d. Interestingly, while the Tyr31αAla mutant abrogated recognition, the Tyr31αPhe 9C1 variant increased the affinity for CD1d–α-GalCer, presumably by reinforcing the hydrophobic character of the 9C1 TCR-CD1d–α-GalCer interface. For the 9B2 TCR, the Gln112βAla mutant resulted in a moderate reduction in affinity, but did not completely ablate binding. This suggests that mutating Gln112β to Ala might enable a compensatory interaction to form via another adjacent residue in the TCRβ chain (for example, Gln108β); or alternatively, that the Gln112βAla mutant of 9B2 is more permissive for binding of endogenous lipid Ags than the WT 9B2 protein, thus resulting in a higher level of autoreactivity. Therefore, contrasting modes of α-GalCer-centric interactions exist between the atypical NKT TCRs and type I NKT TCRs.

## Discussion

Human type I NKT cells are characterized by their expression of the semi-invariant (TRAV10^+^TRAJ18^+^TRBV25-1^+^) TCR and their strong reactivity to α-GalCer presented by CD1d^6^. The type I NKT TCR resembles a pattern recognition receptor in that a universal docking mode underpins type I NKT TCR–CD1d–Ag recognition^8^,^41^. Our findings reveal that CD1d–α-GalCer reactive NKT cells neither have to utilize the semi-invariant TCR, nor do they necessarily have to recognize the resultant CD1d–Ag complex in the consensus type I NKT TCR-CD1d docking topology.

In humans, the type I NKT TCR recognizes a range of chemically diverse lipid Ags by docking over the F′-pocket of CD1d in a parallel manner^42^. Here the invariant TCR α-chain contacts CD1d and the Ag, whereas the TCR β-chain contacts CD1d only. Central to this interaction is the TRAJ18-encoded CDR3α loop, a highly polar loop that makes a number of complementary electrostatic interactions with CD1d–α-GalCer^42^. The importance of the TRAJ18 gene segment for type I NKT cell development is emphasized by the observations that TRAJ18-deficient mice have markedly impaired NKT cell numbers^43^. However, there are exceptions to the use of the invariant TCR α-chain by CD1d–α-GalCer-reactive NKT cells. For example, populations of mouse TRAV13-3^+^TRAJ50^+^ and human TRAV10^−^TRAJ18^+^ NKT cells have been described previously^17^,^22^,^38^. Furthermore, TCR sequencing of human TRAV10^−^ NKT cells showed that while most still expressed TRAJ18, some other TRAJ genes were used in addition to a number of other TCR TRBV genes^21^, although the specificity of these TCRs was not verified. Thus, while variations in the CD1d–α-GalCer-reactive NKT cell repertoire can impact the functional responses and fine specificity towards some Ags, structural analysis of these interactions have nevertheless suggested that they do so under the confines of the consensus footprint on CD1d^8^.

As we had previously described a population of CD1d–α-GalCer-reactive TRAV11^−^TRAJ18^−^ NKT cells in mice^19^, we asked whether such a population of cells could exist in humans. Using α-GalCer, we demonstrated a subset of NKT cells with diverse TCR α and β chain usage. While diverse NKT TCR usage is generally a feature of type II NKT cells, the NKT cells identified here were reactive to the prototypic type I NKT cell Ag, α-GalCer. This meant that these cells could neither be described as type I, nor type II NKT cells, and hence we presently termed that as atypical NKT cells. Notably, they were distinct from the previously described mouse ‘Vα10' (TRAV13-3^+^) NKT subset in that they utilized a diverse array of non-canonical TCRα and TCRβ chain gene segments, therefore suggesting that no apparent mouse homologue of atypical NKT cells has been described. The TRBV25-1^−^ atypical NKT cells were distinct from type I and other, previously defined TRAV10^−^ (but TRBV25-1^+^) non-canonical NKT cells, in that they also did not utilize the TRAJ18 TCR gene segment. Thus, it appears that TRAJ18 and TRBV25-1 are strongly associated with, and may dictate, the archetypal type I NKT cell TCR parallel docking footprint, since in their absence the atypical NKT cell TCRs were able to adopt alternate docking strategies. The basis for the strong association between TRAJ18 and TRBV25-1 expression is unclear, although these TCR elements dominate the interactions with CD1d–α-GalCer in type I NKT TCR complexes. This may indicate that, when used in concert, these TCR motifs preferentially support NKT cell selection criteria during T-cell development, or alternatively, facilitate preferential recognition of a stimulatory sub-class of endogenous Ag. These findings also highlight the fact that diverse TCR usage can also be a feature of CD1d–α-GalCer-reactive NKT cells. Notably, similar to type I NKT cells, these atypical NKT cells could respond to glycolipid Ag presented by CD1d with diverse cytokine production.

Importantly, this repertoire diversity also manifests in differing affinities and functional outcomes towards self- and foreign lipid Ags, in that these atypical NKT TCRs appeared to be of lower affinity to the type I NKT TCRs and also exhibited differing fine specificities. Surprisingly, such differences were attributable to the atypical NKT TCRs adopting a footprint on CD1d that was markedly different to that of consensus F′-pocket docking mode that has consistently been observed for all type I NKT TCRs to date. Namely, two representative atypical NKT TCRs, 9C1 and 9B2, both adopted distinct docking modes above the A′-pocket of CD1d, by binding in an orthogonal manner. These docking modes were reminiscent of the mouse type II NKT TCR (clone XV19) binding to CD1d presenting sulfatide, and moreover, the distribution of contacts across the CDR loops of these atypical NKT TCRs were more analogous to that of type II XV19 NKT TCR recognition^36^,^37^. This A′-pocket docking mode also resonated with the recently described γδ TCR–CD1d–Ag complexes, although naturally the details of the interatomic contacts differed substantially^22^,^44^. In finding different solutions to interact with CD1d, it was interesting to note that molecular mimicry ‘hot spots' underpinned 9C1 and 9B2 TCR recognition. Namely, ‘aromatic motifs' within different regions of the TCRs were seen to play analogous roles in contacting CD1d, despite arising from different regions of the respective TCRs. Furthermore, the atypical NKT cell TCRs also adopted differing strategies to interact with α-GalCer, with interactions via the 6′-OH of α-GalCer featuring prominently in atypical NKT TCR recognition, in stark contrast to typical type I NKT TCR recognition where this motif is not involved in recognition^8^.

Our studies show that the human αβ TCR, δ/αβ TCR and γδ TCR repertoire is sufficiently flexible to recognize the same Ag-presenting molecule displaying the same Ag via a number of different mechanisms. Our findings imply that the TCR repertoire provides significant molecular scope for recognition of diverse lipid-based Ags in the context of CD1d. Given that α-GalCer is being explored as a potential immunotherapeutic agent, and numerous analogues of α-GalCer have been generated to improve the therapeutic efficacy of this drug^8^,^45^,^46^, it is important that we understand the impact of such modifications on the entire CD1d–α-GalCer-reactive NKT TCR repertoire. Our findings have radically reshaped our understanding of NKT TCR recognition.

## Methods

### Accession numbers

The structures of 9B2 TCR–CD1d–α-GalCer, 9C1 TCR–CD1d–α-GalCer and 9C1 TCR were deposited in the RCSB Protein Data Bank (PDB) under the accession codes 4WWK, 4WW2 and 4WW1, respectively.

### Flow cytometry

Blood samples from healthy blood donors were obtained from the Australian Red Cross Blood Service under agreement number 13-04VIC-07, and experiments were conducted in accordance with the University of Melbourne Human Research and Ethics committee guidelines (approval number 1035100). PBMCs were isolated by density gradient centrifugation (Histopaque-1077, Sigma). Cells were stained with CD3ɛ (UCHT1, eBioscience and Becton Dickinson), CD4 (RPA-T4, Becton Dickinson), CD8α (SK1, Becton Dickinson), CD19 (HIB19, BioLegend), CD56 (HCD56, Biolegend), CD69 (FN50, Becton Dickinson), CD158A/B/F/G/H (mixture of DX27, Biolegend, HP-MA4, eBioscience, and UP-R1, eBioscience), CD161 (191B8, Miltenyi Biotec, or HP-3G10, Biolegend), NKG2D (CD314, 1D11, Biolegend), TRAV10 (C15, Beckman Coulter), TRBV25-1 (C21, Beckman Coulter), TRDV1 (A13; a gift from L. Morretta, Istituto Giannina Gaslini, Italy), isotype controls (mouse IgG2b, MPC-11, Biolegend and mouse IgG1, MOPC-21, Biolegend) and 7-aminoactinomycin D viability dye (Sigma). All antibodies were used at empirically determined dilution factors. Cells were stained with human and mouse CD1d tetramers as previously described^22^. CD1d–α-GalCer tetramer^+^ cells were enriched using anti-phycoerythrin magnetic beads (Miltenyi Biotec), followed by cell sorting of CD3^+^ CD1d–α-GalCer tetramer^+^ cells using a FACSAria (BD Biosciences). Cells were then expanded for 14–21 days using anti-CD3, anti-CD28, IL-2, IL-7 and phytohemagglutinin as previously described^22^, and were analysed on an LSRFortessa (BD Biosciences). Data analysis was performed using FlowJo (Tree Star Inc).

### Lipids

C_24:1_ (PBS44) was kindly provided by P. Savage (Brigham Young University). α-GalCer C_26:0_ was supplied by Alexis Biochemicals, and sulfatide (C_24:1_), β-GalCer (C_12_) and β-GlcCer (C_24:1_) were purchased from Avanti Polar Lipids. Disialo-ganglioside GD3 was purchased from Matreya. α-GlcCer (C_20:2_), α-GalCer (C_20:2_ analogue), and OCH were produced in house (at the University of Birmingham, UK). α-GalCer (C_26:0_ 3′,4″-dideoxy- ‘3′-deoxy-α-GalCer' and C_26:0_ 4′,4″-dideoxy ‘4′-deoxy-α-GalCer' analogues) were produced in house (at the University of Connecticut)^47^. Lipids were dissolved in 0.5% v/v Tyloxapol (Sigma), or buffer containing 0.5% v/v tween-20, 57 mg ml^−1^ sucrose and 7.5 mg ml^−1^ histidine, and loaded into CD1d at a three to sixfold molar excess overnight.

### TCR identification

CD3^+^ CD1d–α-GalCer tetramer^+^ γδTCR^−^ TRBV25-1^−^ cells, or alternatively CD3^+^ CD1d–α-GalCer tetramer^+^ TRAV10^−^ cells, were single-cell sorted from CD1d–α-GalCer tetramer-enriched/expanded NKT cells (see above), and complementary DNA generated using SuperScript VILO (Invitrogen) in accordance with manufacturer's instructions. Transcripts encoding TCRα and TCRβ chains were amplified as described^48^, with the exception of 9C1 TCRα, which was identified by 5′-RACE PCR according to manufacturer's instructions (Invitrogen). Here complementary DNA was generated from bulk-sorted CD1d–α-GalCer tetramer^+^ TRBV25-1^−^ cells with a gene-specific TRAC primer (5′-GACCAGCTTGACATCACA-3′), followed by amplification with a nested TRAC reverse primer (5′-GGGAAGAAGGTGTCTTCTGGAAT-3′), and subsequent cloning of PCR products into pGEM-T Easy (Promega). PCR fragments were separated using a 1.5% agarose gel and DNA sequenced by Molecular Diagnostics (the University of Melbourne). TCR sequence analysis was performed using the IMGT online analysis interface, and TCR nomenclature, numbering and CDR3 lengths are presented in accordance with the IMGT system^49^. Unproductively rearranged TCR genes were excluded from analysis.

### Generation of cell lines and stimulation assay

TCR constructs containing full-length TCRα and TCRβ chains separated by a 2A-cleavable linker were synthesized (Genscript), and cloned into the pMIG2 plasmid. Generation of cell lines was achieved by retroviral transduction of αβTCR-deficient Jurkat-76 cells with both TCR and a 2A-cleavable human CD3ɛδγζ construct, using HEK293T cells as packaging cells, essentially as previously described^50^. For stimulation assays, 3 × 10^4^ TCR-expressing Jurkat-76 or SKW3 cells were co-cultured overnight, with or without 3 × 10^4^ C1R (either C1R WT, C1R.CD1d^int^ or C1R.CD1d^hi^) cells, with graded concentrations of lipid in round-bottom 96-well plates, and CD19^−^ cells were analysed by flow cytometry for CD69 expression. For stimulation assays using primary NKT cells, PBMCs were enriched for CD1d–α-GalCer-tetramer^+^ cells using magnetic beads as described above, then CD3^+^ γδTCR^−^ CD1d–α-GalCer tetramer^+^ TRBV25-1^+/−^ cells were enriched by flow cytometric sorting and cultured for 2 days in the presence of plate-bound anti-CD3 (UCHT1, 10 μg ml^−1^), soluble anti-CD28 (CD28.2, 1 μg ml^−1^), IL-2 (100 U ml^−1^), IL-7 (50 ng ml^−1^), PHA (0.5 μg ml^−1^), 10^5^ irradiated allogeneic PBMC and 2 × 10^4^ irradiated CD1d-expressing K562 cells, and subsequently maintained in media containing IL-2 and IL-7. After ∼2 weeks, cultured cells were then re-sorted into type I NKT (CD1d–α-GalCer tetramer^+^ TRBV25-1^+^ TRDV1^−^), atypical NKT (CD1d–α-GalCer tetramer^+^ TRBV25-1^−^ TRDV1^−^), δ/αβ NKT (CD1d–α-GalCer tetramer^+^ γδTCR^−^ TRDV1^+^) and control T (CD1d–α-GalCer tetramer^−^ TRBV25-1^−^ TRDV1^−^) cell subsets, and purity was confirmed (>95%). About 4–5 × 10^3^ cells were cultured with 2 × 10^4^ CD1d-expressing K562 cells, +/−lipid Ag (each at 0.5 μg ml^−1^), for 24 h in 50 μl media containing no IL-2 or IL-7, and cytokine concentrations were assayed by cytometric bead array(BD Biosciences) according to manufacturer's instructions.

### Surface plasmon resonance

SPR experiments were conducted at 25 °C on a ProteOn XPR36 (Bio-Rad) instrument using HBS-T buffer (10 mM HEPES, pH 7.4, 150 mM NaCl and 0.005% surfactant P-20). Biotinylated human CD1d was loaded with α-GalCer (C_26:0_), α-GlcCer (C_26:0_), 3′-deoxy-α-GalCer and 4′-deoxy-α-GalCer, and 400–600 RU was coupled to a GLC sensor chip surface via streptavidin, after which free streptavidin was blocked with an injection of D-biotin. Serial dilutions of purified soluble 9C1 TCR, 9B2 TCR, NKT15 TCR or mutants thereof (starting TCR concentrations between 19.1 and 128 μM) were injected at 25 μl per minute for 60 s, simultaneously over test and control (streptavidin alone) flow cells, using HBS-T buffer. Data were referenced against the control flow cell and analysed using ProteOn Manager version 2.1 (Bio-Rad) software, and *K*_D_, *K*_a_ and *t*_1/2_ values derived using a 1:1 Langmuir binding model. For TCR mutant analysis, *K*_D_ values were normalized against WT TCR *K*_D_ values.

### Generation of soluble TCRs and CD1d

The individual TCRα and β chains of the 9C1 and 9B2 TCRs were synthesized (Integrated DNA Technologies) and cloned into the pET30 vector (Novagen). The 9C1 and 9B2 TCR mutants were produced by overlapping extension PCR with primers that included the desired mutations. The 9C1 and 9B2 wild-type and mutants TCRs were transformed into *E. coli* BL21 (DE3) pLysS for expression and produced as inclusion bodies. Both TCRs were subsequently produced by oxidative refolding as previously described and purified by size exclusion chromatography, hydrophobic interaction chromatography and anion exchange chromatography^51^. Soluble human CD1d either with or without a C terminus BirA biotin ligase tag, along with β2-microglobulin, or mouse CD1d and β2-microglobulin, were cloned into pFastBac Dual (Life Technologies) and expressed by baculovirus infection of High Five insect cell lines as previously described^51^,^52^. CD1d was purified by immobilized metal affinity chromatography followed by size exclusion chromatography using gel filtration (GE Healthcare).

### Structure determination and refinement

The 9C1 TCR-CD1d–α-GalCer and 9B2 TCR-CD1d–α-GalCer complex crystals were obtained in 9–10% PEG 6000/0.1 M MES pH 6.0/4% ethylene glycol and 18% PEG 8000/0.1 M CHES pH 9.5, respectively. The 9C1 and 9B2 complex crystals were flash-frozen and data were collected at the MX2 beamline (Australian Synchrotron) to 2.5 Å and 3.1 Å resolution, respectively. Crystals of the 9C1 TCR were obtained in 20% PEG 3000/0.2 M Na acetate/0.1 M Tris-HCl pH 7.0 and data were collected at the MX1 beamline (Australian Synchrotron) to 1.4 Å resolution. All the data were processed with the programme MOSFLM and were scaled with the CCP4 suite^53^. The 9C1 and 9B2 complex crystals belonged to the C2 and P2_1_2_1_2_1_ space groups, respectively, and the unit cells were consistent with one complex in the asymmetric unit for both complexes. The 9C1 TCR crystal belonged to the P2_1_ space group. For the 9C1 TCR, molecular replacement was carried out with the programme PHASER^54^, using the NKT15 TCR (PDB code: 2PO6). For the 9C1-CD1d–α-GalCer, a molecular replacement solution was found with the programme PHASER^54^ using the structures of human CD1d without the lipid (pdb code: 2PO6) and the refined 9C1 TCR minus the CDR loops as two separate search ensembles. The 9B2 TCR-CD1d–α-GalCer crystal structure was also determined by molecular replacement (PHASER) and using human CD1d without the lipid (PDB code: 2PO6) and the NKT15 TCR minus the CDR loops as two separate search ensembles. For the three crystal structures, an initial run of rigid body refinement was performed with the refinement programme BUSTER 2.10 (ref. 55) and the CDR loops of the TCRs were subsequently rebuilt using the programme COOT^56^. The density of the α-GalCer headgroup was unambiguous for both complexes. After iterative model building with COOT and refinement with BUSTER 2.10, the 9C1 and 9B2 complex structures refinement led to an R/R-free (%) of 20/24.9 and 19.5/25.5, respectively, while an R/R-free (%) of 19.6/21.7 was obtained for the 9C1 TCR structure. The quality of the three structures was confirmed at the Research Collaboratory for Structural Bioinformatics Protein Data Bank Data Validation and Deposition Services website and using the server Molprobity^57^. All presentations of molecular graphics were created with the PyMOL molecular visualization system^58^.

## Additional information

**How to cite this article:** Le Nours, J. *et al.* Atypical natural killer T-cell receptor recognition of CD1d–lipid antigens. *Nat. Commun.* 7:10570 doi: 10.1038/ncomms10570 (2016).

## Supplementary Material

Supplementary Information Supplementary Figures 1-4 and Supplementary Table 1-3

## Figures and Tables

**Table 1:**
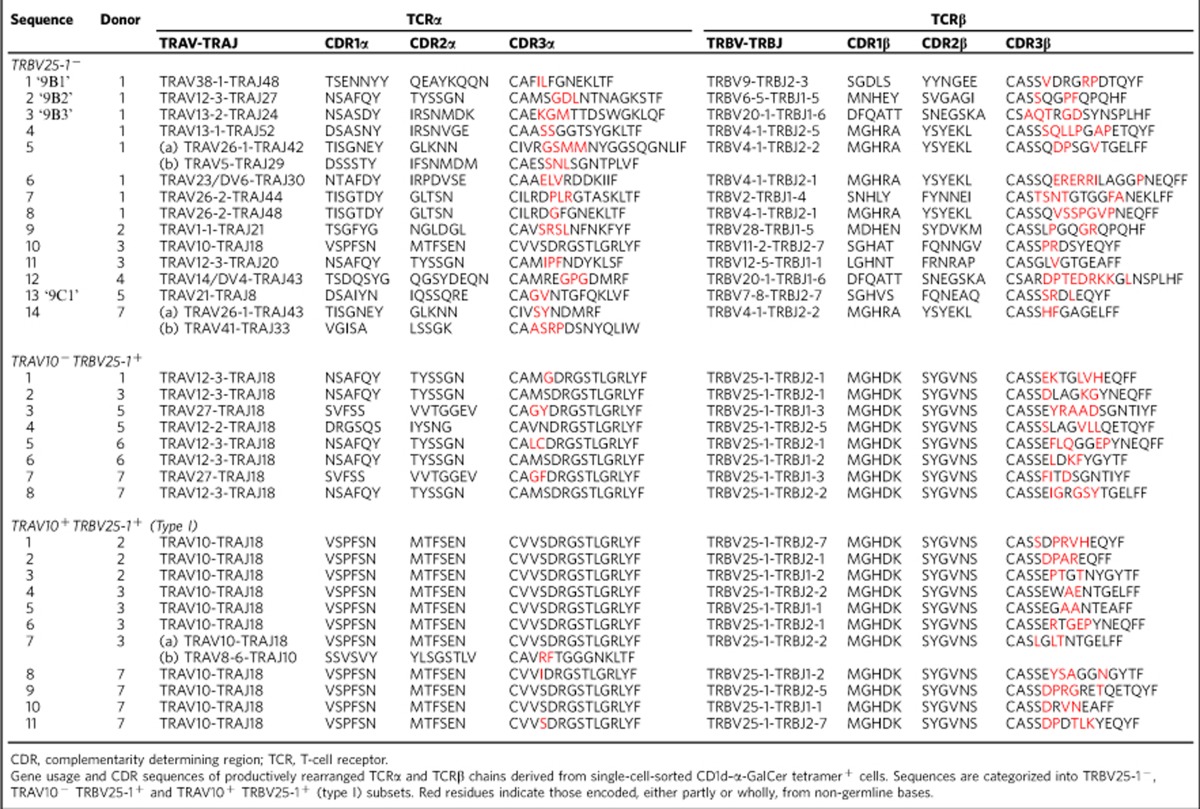
Non-canonical CD1d–α-GalCer-reactive TCR sequences.

**Table 2:** Affinity measurements of 9C1 and 9B2 TCR mutants to CD1d–**α**-GalCer.

**TCR**	***K***_**D**_ **(μM)**
9C1 WT	4
9C1 Tyr31α-Ala	>200
9C1 Tyr31α-Phe	1.9
9C1 Gln112α-Ala	200
9C1 Ser31β-Ala	4.4
9C1 Tyr55β-Ala	180
9C1 Tyr55β-Phe	12
9C1 Gln57β-Ala	17
9C1 Arg109β-Ala	12
9C1 Leu111β-Ala	>200
9B2 WT	4.3
9B2 Gln112β-Ala	7.9

TCR, T-cell receptor.

Binding of soluble 9C1 and 9B2 mutants to CD1d–α-GalCer, as determined by surface plasmon resonance. Dissociation constant (*K*_D_) values for 9C1 WT, Tyr31α-Ala, Gln112α-Ala, Ser31β-Ala, Arg109β-Ala, Leu111β-Ala and 9B2 WT represent the mean of two independent experiments, and 9C1 Tyr31α-Phe, Tyr55β-Ala, Tyr55β-Phe, Gln57β-Ala and 9B2 Gln112β-Ala are derived from a single experiment.
