# Supplementary material for: Atypical natural killer T-cell receptor recognition of CD1d–lipid antigens
Source: Nat Commun. 2016 Feb 15;7:10570. doi: 10.1038/ncomms10570 (PMC4756352; doi:10.1038/ncomms10570)
Supplement: Supplementary Information — Supplementary Figures 1-4 and Supplementary Table 1-3 [file ncomms10570-s1.pdf]

“Atypical Natural Killer T-cell receptor recognition of CD1d-lipid antigens” supplementary Information.

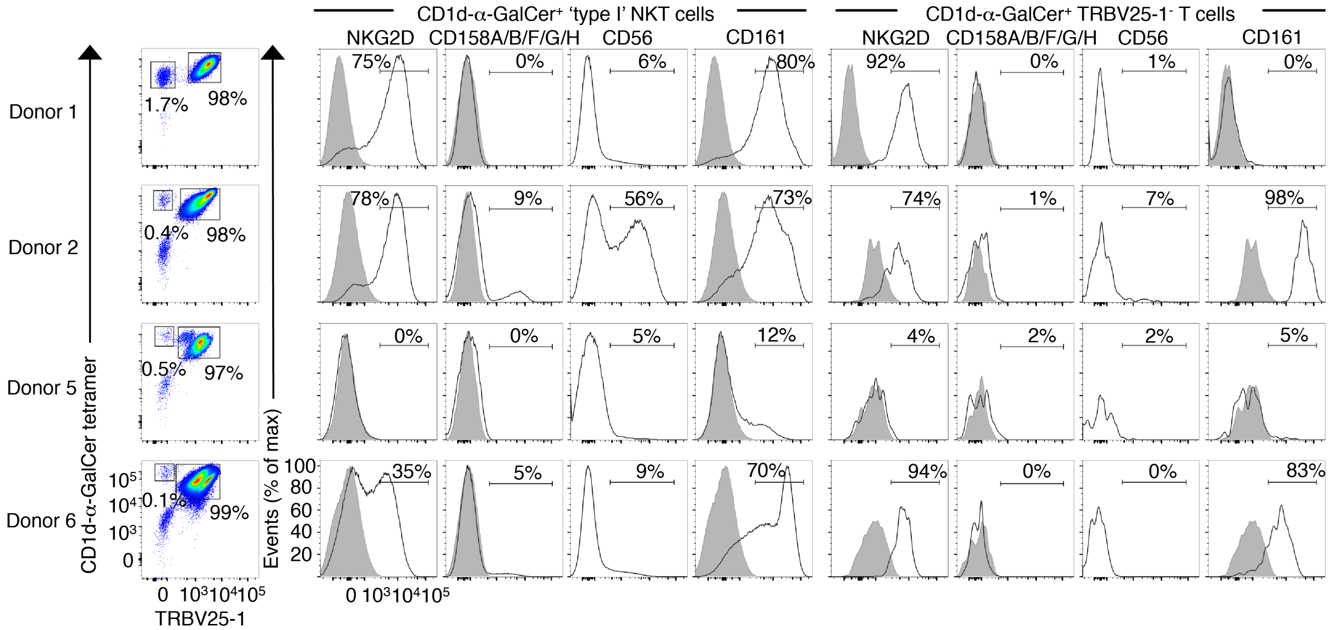

Supplementary Figure 1. Phenotypic analysis of TRBV25-1$^+$ and TRBV25-1$^-$ CD1d-α-GalCer-reactive cells. Four donors with a detectable population of TRBV25-1$^-$ cells after enrichment and *in vitro* expansion of CD1d-α-GalCer tetramer$^+$ TRDV1$^-$ γδTCR$^-$ cells from the blood were analysed for their expression of a panel of cell surface markers. White histograms depict staining for NKG2D, CD158A/B/F/G/H, CD56 and CD161 on TRBV25-1$^+$ ‘type I’ NKT cells (left-hand columns) and TRBV25-1$^-$ cells (right-hand columns), overlayed with isotype controls (gray histograms). Numbers indicate percentage of cells within the indicated gate. Data are representative of two independent experiments for donors 1 and 2, and one experiment for donors 5 and 6.

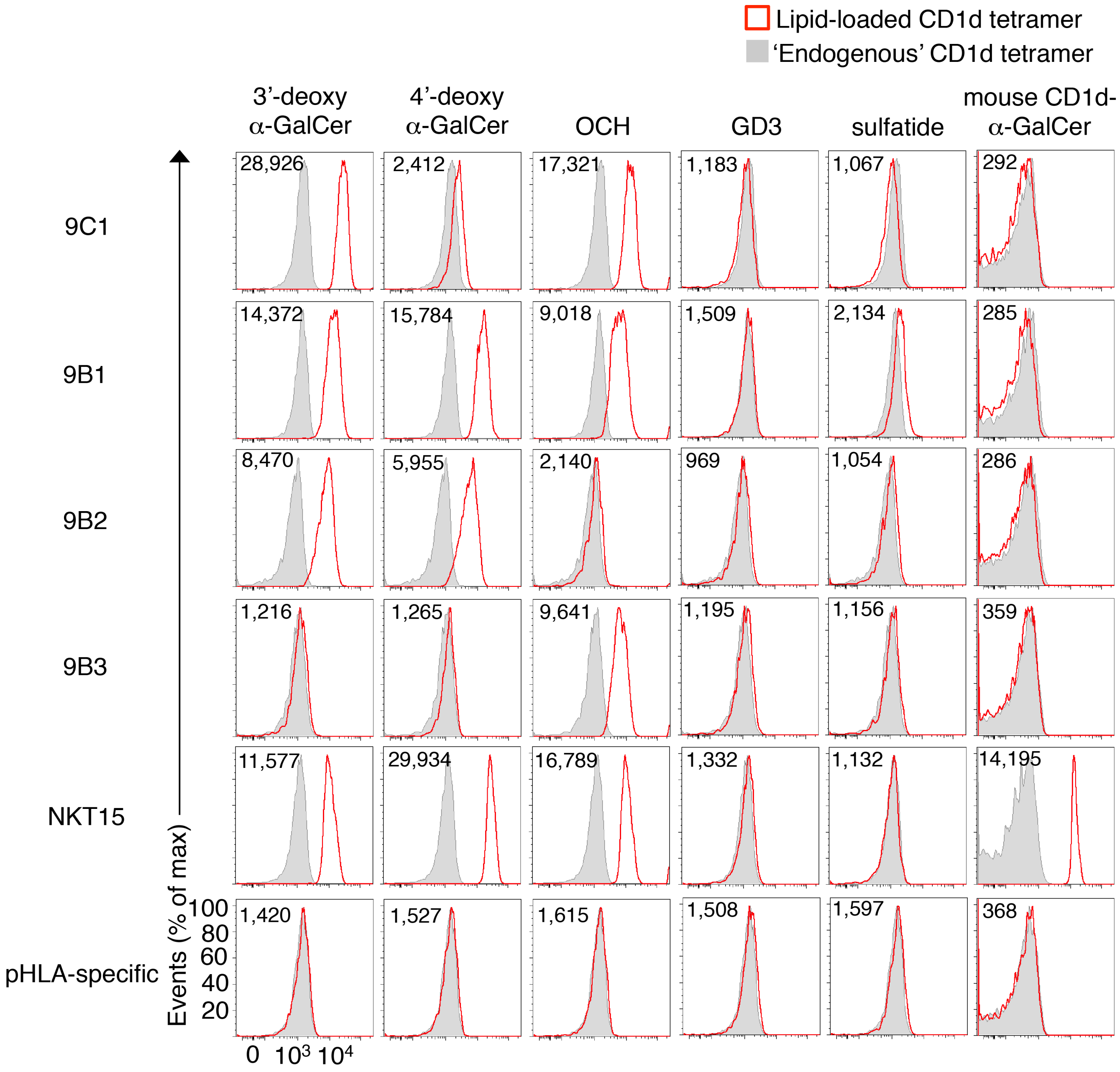

Supplementary Figure 2. CD1d tetramer staining of atypical NKT cell lines. Histograms depicting human CD1d-lipid antigen or mouse CD1d-α-GalCer ($C_{24:1}$ analogue) tetramer staining (red histograms) of $CD3^+$ Jurkat T cell lines transduced with the 9C1, 9B1, 9B2, 9B3 atypical NKT cell TCRs, or with the NKT15 type I NKT cell TCR or an irrelevant pHLA-specific TCR control, overlaid with 'unloaded' tetramers (grey histograms). Numbers in each histogram represent CD1d-lipid tetramer mean fluorescence intensity. Data are representative of one experiment.

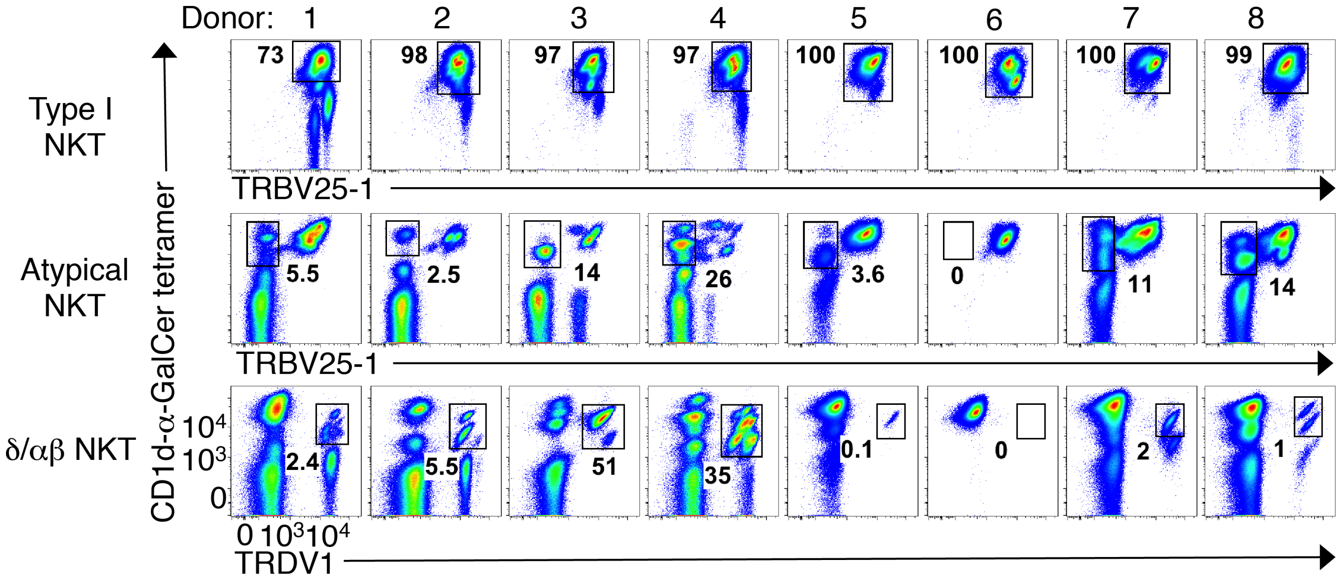

Supplementary Figure 3. Enrichment of Type I, atypical and δ/αβ NKT cells. Density plots show gated subsets of type I (TRBV25-1^{+} TRDV1^{-}), atypical (TRBV25-1^{-} TRDV1^{-}) and δ/αβ (TRDV1^{+}) NKT cell subsets amongst CD1d-α-GalCer tetramer^{+} CD3^{+} γδTCR^{-} cells that were enriched using magnetic beads and cell sorting, and subsequently expanded in vitro. Numbers on plots represent the percentage of gated events. Data shown was performed over 2 independent experiments.

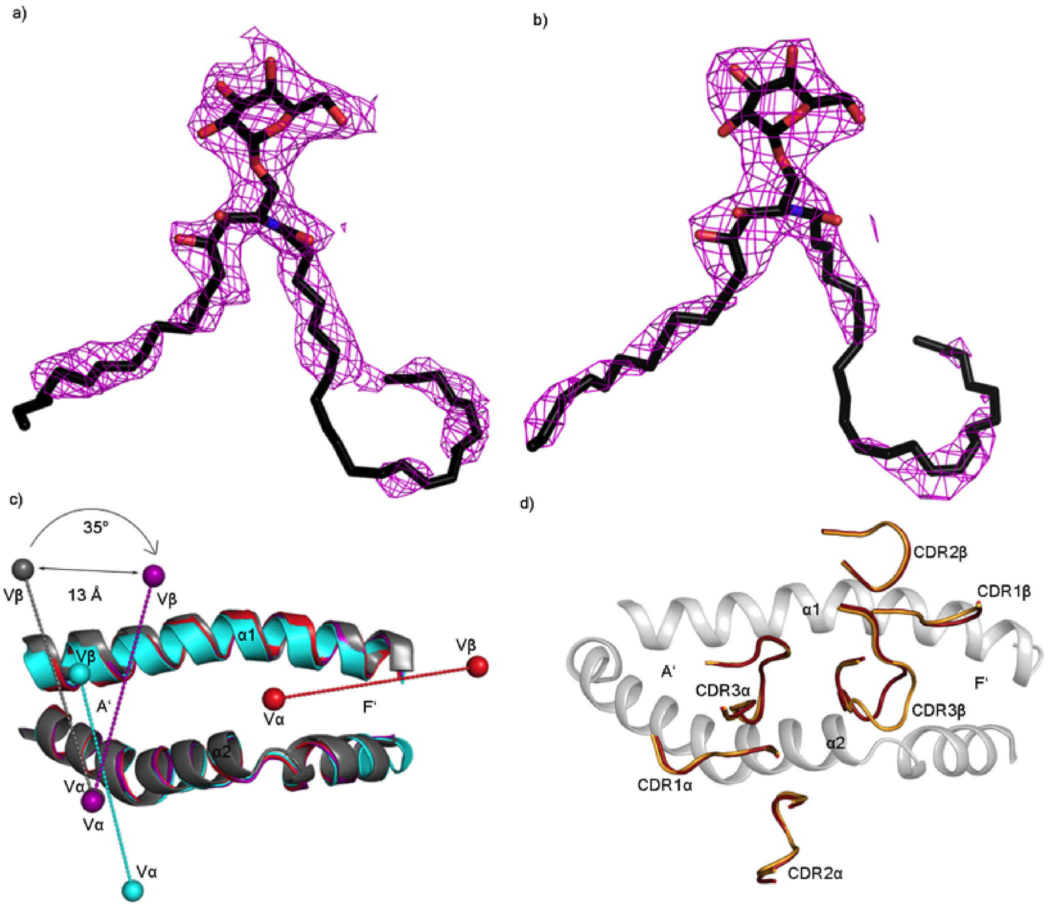

Supplementary Figure 4. Electron density map corresponding to α-GalCer. (**a**) Fo-Fc electron density maps contoured at the 2.5σ level of the lipid antigen α-GalCer in (**a**) the 9C1 TCR-CD1d-α-GalCer ternary complex and (**b**) the 9B2 TCR-CD1d-α-GalCer ternary complex. (**c**) Superposition of the 9C1 TCR-CD1d-α-GalCer (magenta), 9B2 TCR-CD1d-α-GalCer (grey), NKT15 TCR-CD1d-α-GalCer (red) and XV19 TCR-CD1d-sulfatide (cyan) ternary complexes. For clarity, only the α1- and α2- helices of CD1d and the center of mass (spheres) of the respective TCRs are shown. (**d**) Superposition of the unligated 9C1 TCR (in yellow) and the 9C1 TCR in complex with CD1d-α-GalCer (in red). For clarity, only the CDRs are shown.

Supplementary Table 1. Data collection and refinement statistics

	9B2 TCR-CD1d-α-GalCer	9C1 TCR-CD1d-α-GalCer	9C1 TCR
**Data collection**			
Temperature	100K	100K	100K
Resolution limits (Å)	65.37-3.1 (3.27-3.1)	44.80-2.48 (2.58-2.48)	37.28-1.38 (1.45-1.38)
Space Group	$P2_12_12_1$	C2	$P2_1$
Cell dimensions (Å)	*a*=56.4, *b*=76.1, *c*=255.7 α=β=γ=90.00°	*a*=210, *b*=46.3, *c*=124.7 α=γ=90.00° β=120°	*a*=43.1, *b*=73.1, *c*=65.6, α=γ=90.00° β=94.49°
Total N$^{o.}$ observations	73170 (9779)	168213 (36325)	338006 (46559)
N$^{o.}$ unique observations	19363 (2732)	18343 (3953)	83083 (11965)
Multiplicity	3.8 (3.6)	4.6 (4.6)	4.1 (3.9)
Data completeness	94.2 (92.6)	99.5 (97.3)	99.7 (98.8)
Wilson B-factors (Å^2)	61.9	51.8	15.3
I/σ_I	7.5 (2.4)	8.2 (1.6)	9.8 (2.1)
$R_{p.i.m}$ [1] (%)	8.6 (35.6)	4.5 (30.9)	4 (30.4)
**Refinement statistics**			
R_{factor} [2] (%)	19.4	20	19.6
R_{free} [3] (%)	25.6	24.9	21.7
Non hydrogen atoms			
- Protein	6388	6533	3585
- Water	-	170	449
- Heterogen	86	257	-
Ramachandran plot (%)			
- Most favoured	93.95	96	97.65
- Allowed	6.05	4	2.35
Molprobity Score	99th percentile	96th percentile	97th percentile
B-factors (Å^2)			
- Average main chain	64.8	60	17.5
- Average side chain	71.5	67.7	24
rmsd bonds (Å)	0.008	0.008	0.010
rmsd angles (°)	0.94	1.0	1.10

[1] $R_{p.i.m} = \Sigma_{hkl} [1/(N-1)]^{1/2} \Sigma_i | I_{hkl,i} - \langle I_{hkl} \rangle | / \Sigma_{hkl} \langle I_{hkl} \rangle$

[2] $R_{factor} = (\Sigma | |F_o| - |F_c| |) / (\Sigma |F_o|)$ - for all data except as indicated in footnote 3.

[3] 5% of data was used for the R_{free} calculation

Values in parentheses refer to the highest resolution bin.

Supplementary Table 2. 9C1 TCR contacts with α-GalCer and CD1d.

TCR gene	TCR residues	CD1d residues	Bond type
CDR1α	Tyr31	Trp153, Glu156	VDW
CDR1α	Ala29	Trp160	VDW
CDR2α	Ser52	Glu156	VDW
CDR2α	Ser52-Oγ	Glu156-Oε2	HB
CDR2α	Ser52-Oγ	Glu156-Oε1	HB
CDR3α	Thr109	Trp153, Glu156, Thr157, Trp160	VDW
CDR3α	Thr109-O	Thr157-Oγ1	HB
CDR3α	Gly110	His68, Ile69, Trp153, Thr157	VDW
CDR3α	Phe111	Thr65, His68, Trp153	VDW
CDR3α	Phe111-O	Trp153- Nε1	HB
CDR3α	Gln112	His68, Val72, Trp153	VDW
CDR3α	Gln112-Oε1	His68-Nε2	HB
CDR1β	Val30	Arg79	VDW
CDR2β	Gln57-Oε1	Ser76-Oγ	HB
CDR2β	Gln57	Arg79	VDW
CDR2β	Asn58-Oδ1	Arg79-Nη1	HB
CDR2β	Asn58	Arg79	VDW
CDR3β	Leu111	Trp153	VDW
FWβ	Leu66	His68, Arg71, Val72, Ser75	VDW
FWβ	Asp67	His68	VDW
**TCR gene**	**TCR residues**	**α-GalCer atoms**	**Bond type**
CDR1α	Tyr31-Oη	O3A	Water mediated H-bond
CDR3α	Gln112	C6A, O6A	VDW
CDR3α	Gln112-Nε2	O6A	HB
CDR1β	Ser31-Oγ	O4A	Water mediated H-bond
CDR2β	Gln57	C5M, C1A, O5A, C6A, O6A	VDW
CDR2β	Gln57-Nε2	O6A	HB
CDR3β	Arg109	C3A, O3A, C4A, O4A	VDW
CDR3β	Arg109-O	O3A, O4A	HB
CDR3β	Arg109-O	O3A	Water mediated H-bond
CDR3β	Leu111	O4A	VDW
FWβ	Tyr55	O6A	VDW

HB: Hydrogen bond, VDW: Van der Waals, SB: salt bridge. Cut-off at 4 Å for VDW interactions and 3.5 Å for HB. FW, framework.

Supplementary Table 3. 9B2 TCR contacts with α-GalCer and CD1d

TCR gene	TCR residues	CD1d residues	Bond type
CDR1α	Gln31	Trp160	VDW
CDR1α	Tyr32	Trp153	VDW
CDR1α	Tyr32	Trp160	VDW
CDR1α	Tyr32-Oη	Thr157-Oγ1, Trp160-Nε1	HB
CDR2α	Tyr57	Glu156, Trp153	VDW
CDR2α	Ser58	Glu156	VDW
CDR3α	Leu110-O	Asn62-Oδ1	HB
CDR3α	Leu110	Thr165, Leu66, Thr65, Trp160	VDW
CDR3α	Asn111-Nδ2	Gln168-Nε2	VDW, HB
CDR3α	Ala114	Asn62, Gln61	VDW
CDR3α	Ala114-O	Thr65-Oγ1	HB
FWα	Tyr55	Trp153	VDW
FWα	Tyr55-Oη	Trp153-Nε1	HB
FWα	Lys82	Glu156	SB
CDR2β	Ile61	Glu64	VDW
CDR3β	Gly109	His68	VDW
CDR3β	Pro110	His68	VDW
CDR3β	Phe111	Trp160	VDW
CDR3β	Phe111	Ile69	VDW
**TCR gene**	**TCR residues**	**α-GalCer atoms**	**Bond type**
CDR3β	Gln112	O6A	VDW
CDR3β	Gln112	C6A	VDW

HB: Hydrogen bond, VDW: Van der Waals, SB: salt bridge. Cut-off at 4 Å for VDW interactions and 3.5 Å for HB. FW, framework
